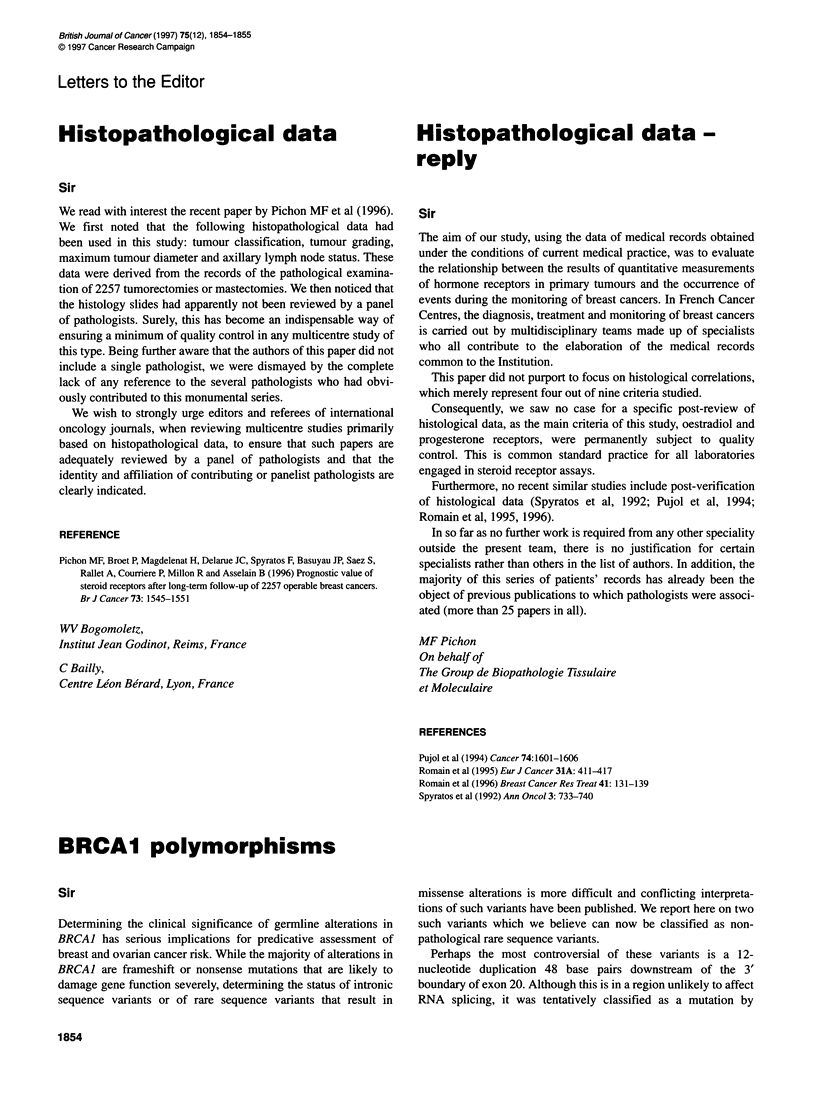# Histopathological data - reply

**Published:** 1997

**Authors:** MF Pichon


### Histopathological data

### Histopathological data – reply

**Sir**

The aim of our study, using the data of medical records obtained under the conditions of current medical practice, was to evaluate the relationship between the results of quantitative measurements of hormone receptors in primary tumours and the occurrence of events during the monitoring of breast cancers. In French Cancer Centres, the diagnosis, treatment and monitoring of breast cancers is carried out by multidisciplinary teams made up of specialists who all contribute to the elaboration of the medical records common to the Institution.

This paper did not purport to focus on histological correlations, which merely represent four out of nine criteria studied.

Consequently, we saw no case for a specific post-review of histological data, as the main criteria of this study, oestradiol and progesterone receptors, were permanently subject to quality control. This is common standard practice for all laboratories engaged in steroid receptor assays.

Furthermore, no recent similar studies include post-verification of histological data (Spyratos et al, 1992; Pujol et al, 1994; Romain et al, 1995, 1996).

In so far as no further work is required from any other speciality outside the present team, there is no justification for certain specialists rather than others in the list of authors. In addition, the majority of this series of patients' records has already been the object of previous publications to which pathologists were associated (more than 25 papers in all).

*MF Pichon*
*On behalf of*
*The Group de Biopathologie Tissulaire*
*et Moleculaire*

### REFERENCES

Pujol et al (1994) *Cancer* **74**:1601–1606
Romain et al (1995) *Eur J Cancer* **31A**: 411–417
Romain et al (1996) *Breast Cancer Res Treat* **41**: 131–139
Spyratos et al (1992) *Ann Oncol* **3**: 733–740